# Dissection of Photosynthetic Electron Transport Process in Sweet Sorghum under Heat Stress

**DOI:** 10.1371/journal.pone.0062100

**Published:** 2013-05-24

**Authors:** Kun Yan, Peng Chen, Hongbo Shao, Chuyang Shao, Shijie Zhao, Marian Brestic

**Affiliations:** 1 Key Laboratory of Coastal Biology & Bioresources Utilization, Yantai Institute of Coastal Zone Research (YIC), Chinese Academy of Sciences (CAS), Yantai, China; 2 State Key Lab of Crop Biology, Shandong Agriculture University, Tai'an, China; 3 Institute for Life Sciences, Qingdao University of Science & Technology, Qingdao, China; 4 Department of Plant Physiology, Slovak University of Agriculture in Nitra, Nitra, Slovakia; 5 The Graduate University of Chinese Academy of Sciences, Beijing, China; George Mason University, United States of America

## Abstract

Plant photosynthesis and photosystem II **(PSII)** are susceptible to high temperature. However, photosynthetic electron transport process under heat stress remains unclear. To reveal this issue, chlorophyll a fluorescence and modulated 820 nm reflection were simultaneously detected in sweet sorghum. At 43°C, J step in the chlorophyll a fluorescence transient was significantly elevated, suggesting that electron transport beyond primary quinone of PSII (Q_A_) (primary quinone electron acceptor of PSII) was inhibited. **PSI** (Photosystem I) photochemical capacity was not influenced even under severe heat stress at 48°C. Thus, PSI oxidation was prolonged and PSI re-reduction did not reach normal level. The inhibition of electron transport between PSII and PSI can reduce the possibility of PSI photoinhibition under heat stress. PSII function recovered entirely one day after heat stress at 43°C, implying that sweet sorghum has certain self-remediation capacity. When the temperature reached 48°C, the maximum quantum yield for primary photochemistry and the electron transport from PSII donor side were remarkably decreased, which greatly limited the electron flow to PSI, and PSI re-reduction suspended. The efficiency of an electron transferred from the intersystem electron carrier **(plastoquinol, PQH_2_**) to the end electron acceptors at the PSI acceptor side increased significantly at 48°C, and the reason was the greater inhibition of electron transport before PQH_2_. Thus, the fragment from Q_A_ to PQH_2_ is the most heat sensitive in the electron transport chain between PSII and PSI in sweet sorghum.

## Introduction

As a result of greenhouse effect, global warming is predicted to persist in the future, and an increased frequency of periods with exceptionally high temperatures is one of the most important characteristics of global warming [1]. Heat stress is generally defined as a rapid and great elevation in ambient temperature [2]. Unlike moderate high temperature stress, a short period of heat stress is enough to provoke severe cellular injury.

Photosynthesis is susceptible to high temperature, and high temperature is liable to impair photosynthetic apparatus in plants [3]. Photosynthetic electron transport from water to NADP^+^ is driven by photosystem II (PSII) and photosystem I (PSI). PSII is highly sensitive to high temperature, and heat-induced injury on PSII certainly can inhibit photosynthetic electron transport [2], [3]. However, it is still unclear about the responses of photosynthetic liner electron transport process, particularly the interaction between PSII and PSI in plants under heat stress. Chlorophyll a fluorescence transient (OJIP transient) has been widely used to study PSII performance in plants under environmental stresses, and it is accepted as a convenient tool to diagnose plant health status. Under high temperature stress, PSII performance usually deceased in plants, and OJIP transient could obviously change [4]–[6]. In recent ten years, a series of studies have clearly revealed the biological meanings of kinetic phases in this transient [7]–[9]. In addition, PSI redox change can be detected by the change in modulated 820 nm reflection, as they significantly correlate with each other [8]. At present, simultaneous detecting OJIP and 820 nm reflection transients served as a feasible way to explore photosynthetic electron transport process and the interaction between PSII and PSI. By using this technique, the effects of chilling and dehydration on photosynthetic electron transport chain were recently reported in apple and cucumber leaves [10], [11], but responses of photosynthetic electron transport chain to heat stress remains to be elucidated.

Sweet sorghum is an annual C4 crop with fast growth rate and high biomass yield. Sweet sorghum is consumed as human food and livestock feed. In addition, it is an important bio-energy crop, as the stalks are rich in fermentable sugars. To date, many studies focus on the procedure of producing bio-energy with sweet sorghum as materials (e.g., [12]–[14]) under the tendency of gradual shrink of ordinary energy source such as coal, oil and natural gas. However, a few of studies pay attention to the relationship between environmental stresses and physiological responses in sweet sorghum, and moreover, these studies mainly associate with salt stress [15]–[17]. To our knowledge, effects of high temperature on sweet sorghum have not been reported. Sweet sorghum has been recognized as a promising crop species for exploiting saline land in coastal zone in China, and air temperature can rapidly rise to extremely high level in summer midday in this region due to low vegetation coverage. Therefore, heat stress studies on sweet sorghum may provide a scientific reference for the practice of saline land exploitation in coastal zone.

Djanaguiraman et al. [18] demonstrated that long-term high temperature (45 days, 40/30°C day/night) reduced photosynthetic rate and PSII photochemical efficiency in the leaves of grain sorghum. However, heat stress on plants may be different compared with long-term high temperature treatment. In this study, we aimed to explore photosynthetic electron transport process in the leaves of sweet sorghum under heat stress by simultaneously examining 820 nm reflection and chlorophyll a fluorescence.

## Materials and Methods

### Plant material and heat treatment

Seeds of sweet sorghum (*Sorghum bicolor* (L.) Moench. cv. YaJin) were immersed in 30°C water for 2 h. Then, fifty seeds were placed in each Petri dish in the dark between two sheets of filter paper at 25°C to germinate, and the filter paper was kept wet by spraying Hoagland nutrient solution (pH 5.7). After 2 days, seeds with similar buds (about 0.6 cm) were transferred to plastic pots filled with vermiculite (one bud in each pot) and grown in artificial climatic chambers (Huier, China). The photon flux density was approximately 200 μmol m^−2^ s^−1^ (12 h per day from 07:00 to 19:00), and day/night temperature and humidity were controlled at 25/18°C and 65%. The seedlings were daily watered with Hoagland nutrient solution (pH 5.7). After 30 days, plants with uniform growth pattern (about 30 cm height and 0.8 cm diameter of the stem) were selected as experimental materials.

Heat stress treatments were conducted in artificial climatic chamber. Seedlings were subjected to 38°C, 43°C and 48°C for 2 h with the light (200 μmol m^−2^ s^−1^, which was equivalent of growth light intensity), and seedlings growing at 25°C were taken as control. Five replicate seedlings were used for each treatment, and the newest fully expanded leaves were used for the following measurements.

### Analysis of photosynthetic rate

Measurement of photosynthetic rate (Pn) was carried out by using an open photosynthetic system (LI-6400XT, Li-Cor, Lincoln, NE, USA) equipped with a LED leaf chamber (6400-02B). Photon flux density was set at 800 μmol m^−2^ s^−1^ in leaf cuvette and the temperature, CO_2_ concentration and relative humidity were not controlled and depended on ambient conditions.

### Measurements of chlorophyll a fluorescence transient and modulated 820 nm reflection

The measurements were conducted by using a multifunctional plant efficiency analyzer (M-PEA, Hansatech, UK). Monitoring modulated reflection change near 820 nm is a very convenient way to follow the redox state of PSI (reaction center + plastocyanin). This instrument was elucidated by Strasser et al. [19] in detail. In this study, leaves were dark adapted for 30 min before they were measured. Dark-adapted leaves were illuminated with 1 s pulse of continuous red light (627 nm, 5000 μmol photons m^−2^ s^−1^) and subsequently, with 10 s far-red light (735 nm, 200 μmol photons m^−2^ s^−1^). Chlorophyll a fluorescence and modulated 820 nm reflection were recorded during the illumination. At the onset of the red light illumination (0.7 ms), PSI (reaction center + plastocyanin) was entirely in reduced state. After the far-red illumination, PSI was completely oxidized. The declined amplitude of modulated 820 nm reflection intensity due to PSI redox change can reflect PSI photochemical capacity [10], [20].

Chlorophyll a fluorescence transients were quantified according to the JIP test by using the following original data: (1) fluorescence intensity at 20 μs (F_o_, when all reaction centers of PSII are open); (2) the maximum fluorescence intensity (F_m_, when all reaction centers of PSII are closed) and (3) fluorescence intensities at 300 μs (K step), 2 ms (J step) and 30 ms (I step). Using these original data, some parameters can be calculated for quantifying PSII behavior [19]. These parameters are listed in Table 1.

**Table 1:** Formulae and terms used in the analysis of the OJIP fluorescence transient.

Data extracted from the recorded fluorensence transient OJIP	
F_t_	Fluorescence intensity at time t after onset of actinic illumination
F_o_ = F_20 μs_	Minimal recorded fluorescence intensity
F_k_ = F_300 μs_	Fluorescence intensity at 300 μs
F_J_ = F _2ms_	Fluorescence intensity at the J step
F_I_ = F_30 ms_	Fluorescence intensity at the I step
F_m_ = F_P_	Maximal recorded fluorescence intensity

Fluorescence parameters derived from the extracted data	
V_t_ = (F_t_–F_o_)/(F_m_–F_o_)	Relative variable fluorescence at time t
V_k_ = (F_k_–F_o_)/(F_m_–F_o_)	Relative variable fluorescence intensity at 300 μs
V_j_ = (F_k_–F_o_)/(F_m_–F_o_)	Relative variable fluorescence intensity at at J step
V_I_ = (F_k_–F_o_)/(F_m_–F_o_)	Relative variable fluorescence intensity at at I step

Biological parameters derived from the fluorescence parameters	
TRo/ABS = 1-F_o_/F_m_	Maximum quantum yield for primary photochemistry
ETo/TRo = 1-V_J_	Probability that an electron moves further than Q_A_
REo/ETo = (1-V_I_)/(1-V_J_)	Probability with which an electron from the intersystem electron carriers is transferred to reduce end electron acceptors at the PSI acceptor side
RC/ABS = M_o_(1/V_J_)(ABS/TRo)	Q_A_ reducing reaction centers per PSII antenna chlorophyll
PI(abs) = RC/ABS·[TRo/(ABS-TRo)]·[ETo/(TRo-ETo)]	Performance index for energy conservation from photons absorbed by PSII to the reduction of intersystem electron acceptors

Q_A_: primary quinone; PSI: photosystem I; PSII: photosystem II.

### Statistical analysis

One-way ANOVA was carried out using SPSS 16.0 (SPSS Inc., Chicago, IL, USA) for all sets of data, and significant differences between means were determined through LSD test. Differences were considered statistically significant when *P*<0.05.

## Results

### Effects of heat stress on relative variable chlorophyll a fluorescence and 820 nm transmission transients

**Figure 1:**
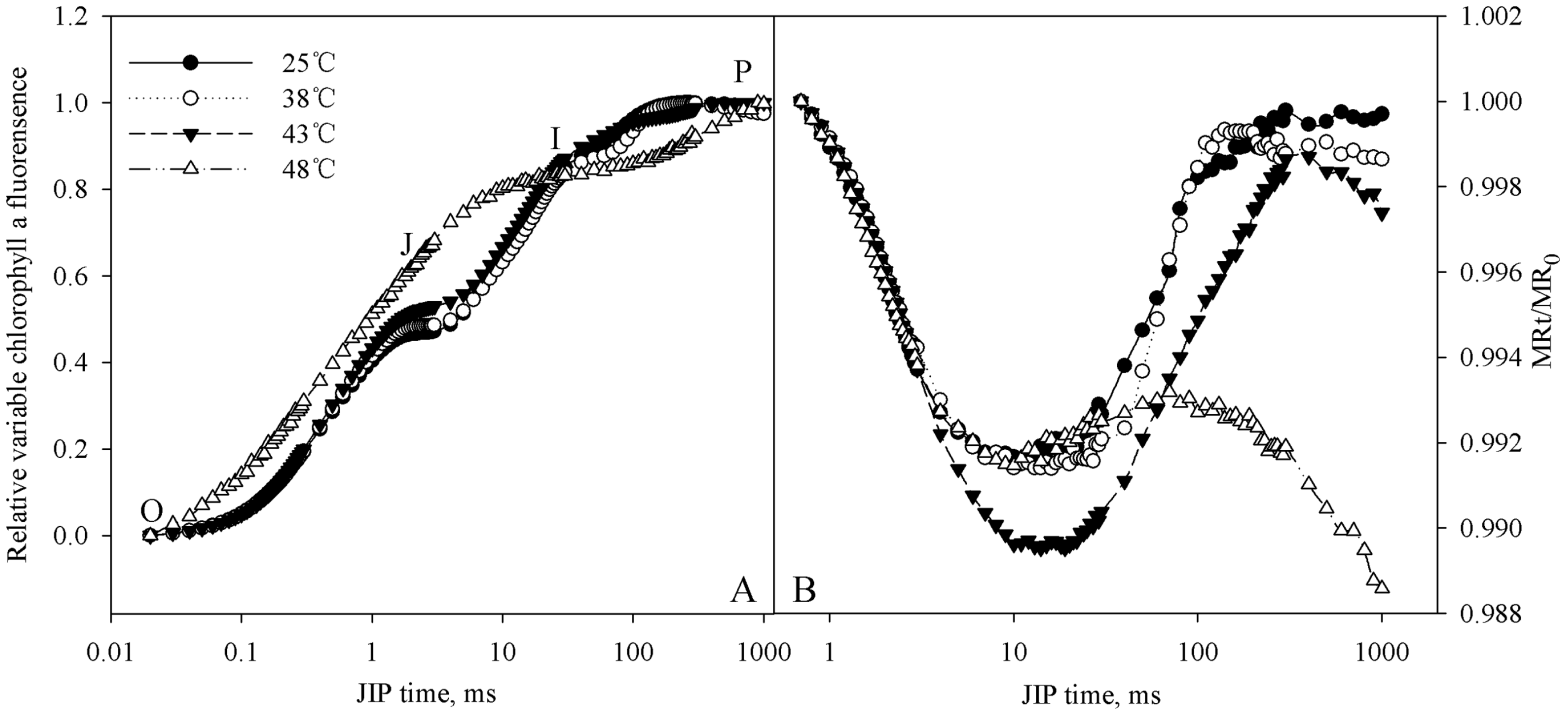
Chlorophyll a fluorescence and modulated 820 nm reflection transients at high temperatures. O, J, I and P indicate the specific steps in chlorophyll a fluorescence transient. The modulated 820 nm reflection signals are presented by MRt/MR_0_ ratio. MRt indicates modulated 820 nm reflection intensity at time t. MR_0_ is the value at the onset of actinic illumination (at 0.7 ms).

As shown in Fig. 1, chlorophyll a fluorescence and 820 nm reflection transients were not affected by heat stress at 38°C. J step was significantly elevated at 43°C, and chlorophyll a fluorescence transient changed greatly at 48°C (Fig. 1A).

The modulated 820 nm reflection signals are presented by MRt/MR_0_ ratio (Fig. 1B). MRt indicates modulated 820 nm reflection intensity at time t, and MR_0_ is the value at the onset of actinic illumination (at 0.7 ms). Decrease in MRt/MR_0_ from MR_0_ (at 0.7 ms) to the minimal value (at about 12 ms) reflects PSI oxidation process. The minimal value point is a transitory steady state with equal oxidation and re-reduction rate of PSI. Subsequently, increase in MRt/MR_0_ indicates PSI re-reduction. PSI oxidation amplitude increased at 43°C, and the following re-reduction did not reach the normal level at 25°C (Fig. 1B). Under the severe stress at 48°C, 820 nm reflection transient changed greatly, and PSI re-reduction nearly suspended (Fig. 1B).

### Effects of heat stress on photosynthetic rate, PSII performance and PSI photochemical capacity

**Figure 2:**
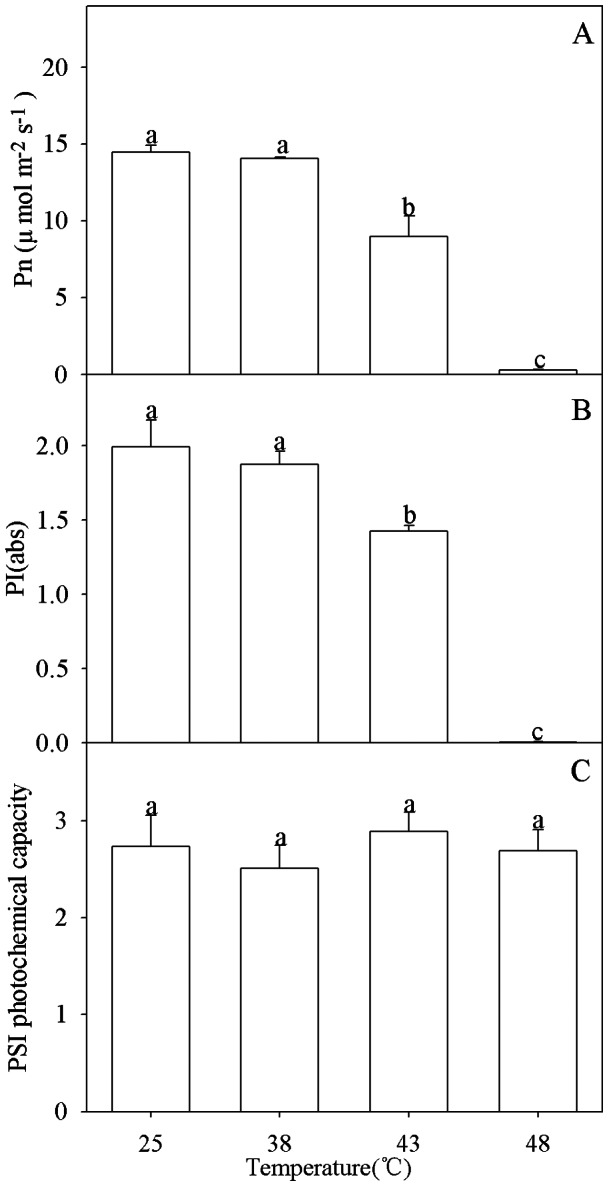
(A) Photosynthetic rate (Pn), (B) PSII performance index (PI(abs)) and (C) PSI photochemical capacity at high temperatures. Data in the figure indicate mean of five replicates (± SD). Different letters on error bars indicate significant difference at *P*<0.05.

Pn and PI(abs)(PSII performance index on absorption basis) were significantly decreased by heat stress at 43°C (*P*<0.05), and the decrease become greater at 48°C (*P*<0.05) (Fig. 2A and B). In contrast, PSI photochemical capacity was not inhibited by heat stress even at 48°C (Fig. 2C).

### Effects of heat stress on PSII behaviors

**Figure 3:**
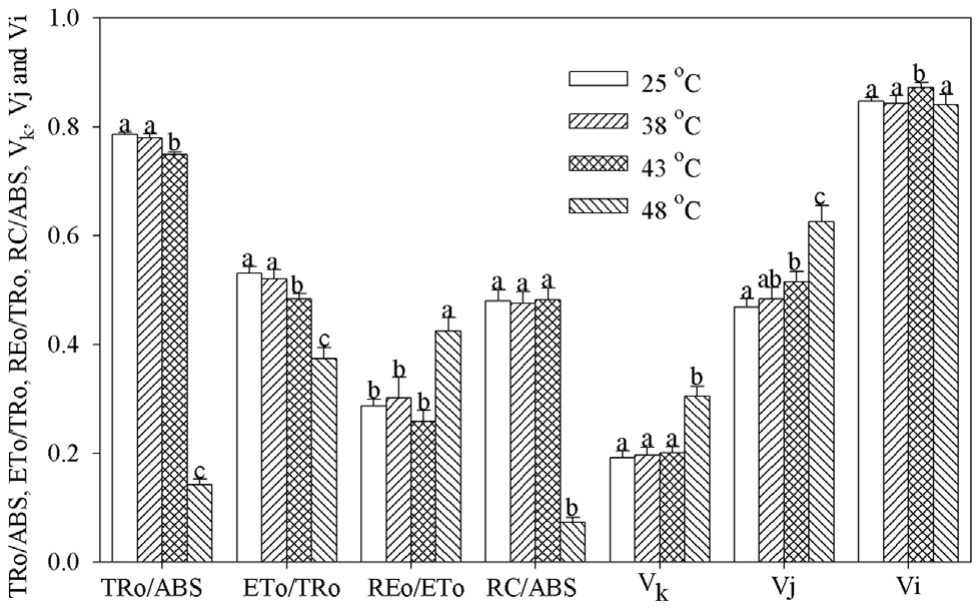
TRo/ABS, ETo/TRo, REo/ETo, RC/ABS, V_k_, Vj and Vi at high temperatures. The definition for these parameters is in Table. 2. Data in the figure indicate mean of five replicates (± SD). Different letters on error bars indicate significant difference at P<0.05.

At 48°C, RC/ABS significantly decreased (*P*<0.05), whereas V_k_ (relative variable fluorescence intensity at 300 μs)increased significantly (*P*<0.05) (Fig. 3). Vj and Vi remarkably increased at 43°C (*P*<0.05), whereas TRo/ABS(maximum quantum yield for primary photochemistry) and ETo/TRo (probability that an electron moves further than Q_A_)significantly decreased (*P*<0.05), and the decrease became greater at 48°C (Fig. 3).

**Table 2:** Recovery of PI(abs), TRo/ABS, V_k_, Vj, Vi and RC/ABS in the leaves one day after heat stress at 43°C and 48°C for 2 h.

Parameters	Control (25°C)	43°C for 2 h	48°C for 2 h
PI(abs)	1.99±0.18a	1.92±0.20a	0.0066±0.0020b
TRo/ABS	0.79±0.0034a	0.78±0.0013a	0.14±0.020b
V_k_	0.19±0.012a	0.20±0.013a	0.35±0.038b
Vj	0.47±0.013a	0.47±0.019a	0.72±0.050b
Vi	0.85±0.015a	0.84±0.013a	0.93±0.047b
RC/ABS	0.48±0.021a	0.47±0.012a	0.072±0.0070b

### Recovery of photosynthetic apparatus one day after heat stress

PI(abs), Vj(relative variable fluorescence intensity at 2 ms), Vi(relative variable fluorescence intensity at 30 ms), TRo/ABS and ETo/TRo recovered to the normal level (25°C) one day after heat stress at 43°C for 2 h (Table. 2). However, no recovery was observed in PI(abs), TRo/ABS, ETo/TRo, V_k_, Vj, Vi and RC/ABS (Q_A_ reducing reaction centers per PSII antenna chlorophyll)one day after the severe heat stress at 48°C (Table. 2), and the leaves became curly and parching and tended to die.

## Discussion

Photosynthesis and PSII were negatively affected by heat stress in sweet sorghum, as a significant decrease in Pn and PI (abs) was noted (Fig. 2A and B). In contrast, PSI photochemical capacity was not influenced by heat stress, suggesting higher heat tolerance in PSI than PSII (Fig. 2C). Heat sensitivity of photosynthesis and PSII has been extensively reported in other crops in previous studies [21]–[23]. However, a few studies demonstrated that PSI was more heat tolerant than PSII in *Triticum aestivum*, *Spinacia oleracea*, *Haberlea rhodopensis* and *Arabidopsis* [24]–[27]. This study on sweet sorghum further confirmed the heat resistance of PSI.

When temperature rose to 43°C, PSI oxidation amplitude increased and PSI re-reduction could not reach the normal level in the first 1 s red illumination (Fig. 1B). The reason was mainly attributed to the heat-induced decrease in electron donation from PSII, and in other words, the imbalance between PSII and PSI appeared. J step suggests the kinetic bottlenecks of the electron transport chain resulting in the momentary maximum accumulation of Q_A_^−^ [8], and I step also represents the subsequent kinetic bottlenecks of the electron transport chain but due to the limitation of plastoquinol (PQH_2_) re-oxidation [9]. Both J and I steps were elevated significantly at 43°C, suggesting that electron transport beyond Q_A_ and beyond PQH_2_ were both inhibited, and consequently, ETo/TRo decreased significantly, however, REo/ETo(probability with which an electron from the intersystem electron carriers is transferred to reduce end electron acceptors at the PSI acceptor side.) did not change at this temperature and even increased greatly at 48°C (Fig. 3). REo/ETo depends on the electrons transferred to PSI from PQH_2_ and the electron influx from upper electron carrier. Heat-induced increase in REo/ETo resulted from the less electrons donated to reduce PQH_2_. Thus, the electron transport chain beyond PQH_2_ is less sensitive to heat stress than that from Q_A_ to PQH_2_ in sweet sorghum.

Oxygen-evolving complex (OEC) is considered as the most heat sensitive component of PSII [3]. Increase in V_k_ is a specific indicator for heat-induced damage to OEC [28], [29]. No significant change in V_k_ at 43°C (Fig. 3) indicated that OEC was not affected and the electron transport from PSII donor side to PSII reaction center was not inhibited. In disagreement with previous studies [4], [30], the above results illustrate that electron transport chain of PSII acceptor side is more susceptible to heat stress compared with PSII donor side in sweet sorghum. In our opinion, the conflict derives from different treatment protocol. Heat treatment was conducted in dark in previous studies, whereas heat stress in this study was performed under growth light intensity in order to make the heat treatment more physiologically relevant. Yang et al. [29] pointed out that the mechanisms of PSII inactivation were different under heat stress with and without light. High light damaged the PSII acceptor side more severely than the PSII donor side [31]. Chen et al. [32] proved that high temperature combined with light damaged the acceptor side of electron transport chain to a greater degree than high temperature alone in apple peel. Therefore, it is conceivable that electron transport in PSII acceptor side was earlier inhibited in this study. PSI photoinhibition is more dangerous than PSII photoinhibition because of the very slow recovery rate of PSI [33]. PSI photoinhibition is mainly induced by reactive oxygen species produced at the acceptor side of PSI through Mehler reaction in vivo [34]. Thus, electron flow from PSII is responsible for PSI photoinhibition, and the addition of 3-(3,4-dichlorophenyl)-1,1-dimethylurea, an inhibitor of Q_A_ oxidation, can completely suppress PSI photoinhibition and help PSI recovery after chilling stress [11], [35]. Carbon fixation process has been proved highly susceptible to heat stress [36], which can reduce the NADPH production through linear electron transport, and in consequence, the possibility of PSI photoinhibition may rise due to more electrons transferred to Mehler reaction. Therefore, decrease in ETo/TRo at 43°C could help to reduce the possibility of PSI photoinhibition by limiting the production of reactive oxygen species from Mehler reaction in this study. Thus, we suppose that decrease in ETo/TRo is a self-protection strategy in sweet sorghum at 43°C. RC/ABS was not affected at 43°C (Fig. 3), suggesting that the effective antenna size and active reaction centers were not affected. The significant decrease in TRo/ABS at 43°C indicated that PSII photoinhibition occurred in sorghum (Fig. 3). The declined TRo/ABS also could be considered as positive adaption for down-regulating the photosynthetic excited pressure, and it might be induced by the photo-protective mechanisms including non-photochemical dissipation or state transition [37]–[39]. The photosynthetic proteins and lipids might not be greatly affected at this temperature. PSII function recovered entirely one day after heat stress at 43°C (Table. 2), implying that sweet sorghum has certain capacity to protect itself against heat stress through physiological regulation.

When temperature reached 48°C, OEC and PSII reaction center were damaged, as increase in V_k_ and decrease in RC/ABS were greatly significant (Fig. 3). As a result, photosynthetic electron donation to PSI was sharply lowered, and PSI re-reduction became impossible (Fig. 1B). One day after the stress, the leaves could not recover and tended to die. The inreversible damage on PSII at this temperature should result from heat-induced protein denaturation and lipid oxidation.

In conclusion, PSI photochemical capacity was not affected by heat stress in sweet sorghum. Electron transport of PSII acceptor side was initially inhibited by heat stress, and the fragment from Q_A_ to PQH_2_ is the most heat sensitive in the electron transport chain between PSII and PSI. The decrease in electron transport between PSII and PSI may play a self-protection role in reducing the possibility of PSI photoinhibition.
